# Application of Max-SAT-based ATPG to optimal cancer therapy design

**DOI:** 10.1186/1471-2164-13-S6-S5

**Published:** 2012-10-26

**Authors:** Pey-Chang Kent Lin, Sunil P Khatri

**Affiliations:** 1 Department of ECE, Texas A&M University, College Station, TX 77843, USA

## Abstract

**Background:**

Cancer and other gene related diseases are usually caused by a failure in the signaling pathway between genes and cells. These failures can occur in different areas of the gene regulatory network, but can be abstracted as faults in the regulatory function. For effective cancer treatment, it is imperative to identify faults and select appropriate drugs to treat the faults. In this paper, we present an extensible Max-SAT based automatic test pattern generation (ATPG) algorithm for cancer therapy. This ATPG algorithm is based on Boolean Satisfiability (SAT) and utilizes the stuck-at fault model for representing signaling faults. A weighted partial Max-SAT formulation is used to enable efficient selection of the most effective drug.

**Results:**

Several usage cases are presented for fault identification and drug selection. These cases include the identification of testable faults, optimal drug selection for single/multiple known faults, and optimal drug selection for overall fault coverage. Experimental results on growth factor (GF) signaling pathways demonstrate that our algorithm is flexible, and can yield an exact solution for each feature in much less than 1 second.

## Background

In all organisms, cell function is supported by the interaction of genes and protein products, forming an interconnected network called the gene regulatory network (GRN) [1]. The interaction or communication between genes and cells is highly complex and multivariate. Cancer and gene-related diseases are often the result of a failure in the signaling, leading to incorrect gene regulation and its associated functions.

The modeling of the gene interactions is thus highly important for understanding the mechanism and therapy of cancer. Because genes are observed to have a switch-like expression (active or inactive), the Boolean network model [2] has become popular for representing the GRN. In the Boolean network, the genes and biochemical pathways are represented as logic functions, much like logic gates in an integrated circuit (IC). This network can be extended to include signaling failures and defects in the GRN, which are represented as faulty lines in the circuit [3]. The issue of faults in circuits is well understood in electronic testing. For example, in chip manufacturing, circuits are typically tested to check that the IC is defect free before vendoring. Manufacturing defects manifest themselves as logical faults modeled as lines (wires) stuck-at '1' or '0'. Using this *stuck-at fault model*, automatic test pattern generation (ATPG) algorithms determine a set of tests (bit vectors on the inputs of the circuit) to test for stuck-at faults in the circuit.

In this paper, we use the stuck-at fault model for the GRN [3] and employ ATPG techniques to determine a drug vector (set of drugs) to rectify the fault. The ATPG algorithm is developed as a Boolean satisfiability (SAT) based method, where the Boolean network is transformed into a conjunctive normal form (CNF) expression and solved for satisfiability to find the drug vector. In therapy, the goal is to treat the cancer (represented by one or more faults) using drugs with the least negative impact on the patient, ideally by prescribing the fewest number of drugs necessary to avoid unnecessary side-effects and cost. The SAT method is further extended by assigning weights to the circuit outputs and drug vectors, and solved with a weighted partial Max-SAT to find the optimal set of drugs to fix or rectify the fault.

The key contributions of this paper are:

• In contrast to previous approaches [3] which performs an explicit search, we develop an implicit SAT-based ATPG approach to model and identify detectable faults (single and multiple) in a Boolean network.

• By assigning weights to model output and drug vectors, we use a weighted partial Max-SAT formulation to determine the optimum selection of drugs to rectify a specific fault.

• Our approach can be trivially extended to handle multiple faults.

• We utilize the above techniques for drug therapy to select the minimum set of drugs to provide the best coverage across all single/multiple faults.

The remainder of this paper is organized as follows. The next section discusses previous work in this area. In the following section, we introduce fault-modeling and Boolean satisfiability and describe our approach for drug therapy. We then present experimental results obtained from applying our methods to a biological example and discuss applications of our algorithm towards sequential circuits. Lastly, we draw some concluding remarks about our SAT-based ATPG method.

## Previous work

In the actual GRN, the gene expression or protein concentration is continuous. However, in this paper, the Boolean network (BN) [2] is chosen as preferred network for modeling the GRN. There are several reasons for this choice. First, it has been observed that many genes exhibit a switch-like ON/OFF activity in terms of their expression [4]. Second, a discrete model like the BN is relatively simple and easy to analyze and simulate. And lastly, there are many logic synthesis and test algorithms already developed in circuit design and testing that can be applied to the Boolean network.

In [3], the authors proposed modeling cancer as faults in the signaling network and applied fault analysis for drug intervention to control the GRN. Cancer is a disease that arise from fault(s) in the network leading to loss of cell cycle control and uncontrolled cell proliferation. Therapy involves both identification of the fault and a suitable drug combination to target the fault. This paper focused on the growth factor (GF) signaling pathways, which are often associated with proliferation of cancer. The GRN is modeled using Boolean logic gates and all possible single faults are enumerated. All drug combinations were also simulated to determine the effectiveness of drug combinations towards each fault.

The method proposed in [3] is an ATPG technique in principle. Our approach is similar to [3] in that it uses the BN and models cancer as faults in the network. However, the differences are several. Instead of explicit enumeration of the BN, we use an extensible, implicit SAT-based ATPG approach to efficiently model and identify faults, and perform drug selection. Further, unlike [3], we include weighted clauses for outputs and drugs in the SAT formulation. Using this, the algorithm can implicitly and efficiently determine the drug combination which is maximally effective. Finally, our approach can handle multiple faults easily. The runtimes of our approach are typically much less than a second per set of faults.

In the past, ATPG has been extensively studied in research and industry. One such ATPG technique is the SAT-based ATPG [5-7] which translates the testing condition into a SAT instance that retains the circuit structure. A test for the fault can then be found by invoking a SAT solver. In the context of cancer therapy, we extend the SAT based approach to handle drugs and multiple faults.

SAT-based approaches have been applied to the analysis of GRNs and Boolean networks. Assuming an asynchronous logical description of the GRN, [8] presents an approach for expressing GRN constraints into a Boolean formula, from which they infer parameters of the GRN. While in [9], an algorithm is presented to find all attractors in a Boolean network based on a SAT-based bounded model checking. This algorithm uses a SAT-solver to identify paths of a particular length in the state-transition graph of a Boolean network. In these previous works, SAT has been used to infer the GRN. This fundamentally differs from our work which uses SAT to simulate the faulty GRN and control the GRN using drugs.

Control of Boolean networks has been studied from a theoretical standpoint in [10] and using a model checking algorithm in [11]. In these papers, a BN with control nodes is given, and the control strategy denotes a sequence of control signals that deterministically drive the BN from a given initial state, to a desired final state, in *t *time steps. Conceptually, our SAT-based ATPG approach is similar to these methods of Boolean network control, in that we construct a SAT formula to check whether a selection of drugs can drive the system to a desired state. However we differ in a few key areas. First, our approach considers the BN under a stuck-at fault model, in that one or more of the genes can be faulty. This model allows us to apply ATPG techniques to identify faulty genes in the BN which can lead to undesired GRN behavior. And secondly, our approach weighs the drugs and outputs in the ATPG formulation, allowing for different control strategies depending on desired specifications (i.e. selection with fewest drugs or fewest side effects). Unlike [10,11], our method can also determine the best drug selection on a BN where the faulty gene location is unknown.

## Method

In this section, we present our SAT-based ATPG method. Before the method is described in detail, we first provide definitions for fault modeling and Boolean Satisfiability.

### Fault terminology

A manifestation of a defect at the abstracted function level is called a **fault**.

In an IC, the difference between a defect and a fault can be explained as imperfections in the hardware and function, respectively. While in genomics, examples of biological defects can include mutations in the gene activation site, malformation of the protein folding, and problems in the gene product transport. Likewise, an example of a biological fault is a modification of the logical function representing a gene, producing the incorrect output. A **stuck-at fault **is modeled by assigning a fixed (0 or 1) value to a signal line (input or output of a logic gate) in the circuit.

An **untestable fault **is a fault which no test can detect. Untestable faults appear in two situations.

• Faults that are *redundant*, whose presence does not change the output behavior of the circuit.

• Faults that change the output behavior of the circuit, but no test (drug vector in the context of cancer therapy) can be generated to propagate or rectify the fault.

### Stuck-at fault modeling

In the Boolean network model for a GRN, the activity of genes is modeled as a Boolean circuit. We assume the circuit is modeled as an interconnection of Boolean gates. A stuck-at fault is assumed to only affect interconnections (wires or nets) between gates. Each net can have one of two types of faults: stuck-at-1 or stuck-at-0 (s-a-1 and s-a-0, respectively). Thus, a net with a stuck-at-0 fault will always have a logic value 0, irrespective of the correct logic output of the gate (gene) driving the net.

**Figure 1:**
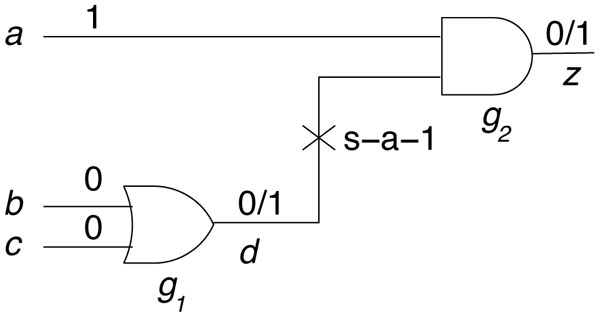
**Circuit with stuck-at fault**.

As an example, consider the circuit of Figure 1 comprising of an OR gate driving an AND gate. Also consider a stuck-at-1 fault at the output of the OR gate, which means that the faulty line remains 1 irrespective of the input state of the OR gate. If the normal (good) output of the OR gate is 1 (in the case where its inputs were *< bc *> = 01,10,11), then this fault will not affect any signal in the circuit. However, the input <*bc *> = 00 to the OR gate should produce a 0 output in the good circuit. The good (faulty) value 0 (1) is applied to the AND gate. If the input vector <*abc *> = 100, the good circuit output (true response) and faulty output would differ. Hence <*abc *> = 100 is called a test for the s-a-1 fault on the output of the OR gate.

**Figure 2:**
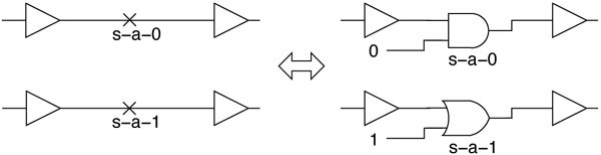
**Fault modeling and injection**.

A stuck-at-0 fault is modeled by inserting a two-input AND gate at the fault site as shown in Figure 2. The side input of the gate is driven by a signal which is set to 1 to simulate a fault-free site, or set to 0 to inject the s-a-0 fault. Similarly, the circuit with a s-a-1 fault is modeled by inserting an OR gate at the site. The side input of this OR gate is set to 0 to simulate a fault-free site, or set to 1 to inject the s-a-1 fault. These gates are inserted at every net (wire), allowing the simulator to inject faults at any site.

Note that drugs are modeled the same as stuck-at faults, wherein a drug that inhibits a gene is modeled as a s-a-0 "fault", while a drug that activates a gene is modeled as s-a-1 "fault". The gates for drug injection are inserted at the nets of the genes that they target.

### Boolean satisfiability

Several ATPG algorithms [5-7], including the method proposed in this paper are based on Boolean Satisfiability (SAT) and utilize the stuck-at fault model. We begin with an overview of SAT, followed by a SAT-based formulation of the ATPG problem.

A **literal **or a **literal **function is a binary variable *x *or its negation $\bar{x}$.

A **clause **is a disjunction (logical OR) containing literals (example: $\left(x+ȳ+\bar{z}\right)$).

A **Conjunctive Normal Form (CNF) **expression *S *consists of a conjunction (AND) of *m *clauses *c*_1 _... *c_m_*. Each clause *c_i _*consists of disjunction (OR) of *k_i _*literals. A CNF *S *is **satisfied **if it evaluates to 1. Satisfying *S *is equivalent to satisfying all *c_i _*∈ *S*

Given a Boolean formula *S *(on a set of binary variables *X*) expressed in CNF, the objective of SAT is to identify an assignment of the binary variables in *X *that satisfies *S*, if such an assignment exists.

For example, consider the formula $S\left(a,b,c\right)\;=\;\left(a+\bar{b}\right)\cdot \left(a+b+c\right)$. This formula consists of 3 variables, 2 clauses, and 4 literals. This particular formula is satisfiable, and a satisfying assignment is (*a*,*b*,*c*) = (0,0,1) or $ā\bar{b}c$. There are several extensions to the SAT problem. One such extension of interest is **All-SAT**. For a SAT formula, there may exist many satisfying assignments. The objective of All-SAT is to find *all *satisfying assignments. Another useful extension is **Weighted partial Max-SAT **(WPMS) which aims to satisfy a partial set of clauses. In WPMS, each clause in the CNF is identified as a hard clause or soft clause. Each soft clause is associated with a weight. The problem then is to identify an assignment that satisfies *all *hard clauses while maximizing the total weight of the satisfied soft clauses.

### SAT-based formulation for stuck-at fault model

In the SAT based ATPG method, we first generate a formula in CNF to represent tests for the fault. To do so, the circuit from the stuck-at fault model must be converted to a CNF. Every gate (*g_i_*) of the circuit has CNF formula (*G_i_*) associated with it, which represent the function performed by the gate. The formula is true iff the variables representing the gate's inputs and outputs take on values consistent with its truth table.

For example, consider a 2-input AND gate (*g_j_*) with the lines *x *and *y *as inputs and *z *as output. The CNF formula (*G_j_*) for the AND gate is written as:

$$G_{j}=\left(\bar{z}+x\right)\cdot \left(\bar{z}+y\right)\cdot \left(z+\bar{x}+ȳ\right)$$

A CNF formula for the entire circuit *S *is obtained by forming the conjunction of the CNF formulas for all the gates of the circuit. If there are *n *gates in the circuit, then the CNF formula *S *for the entire circuit is written as:

$$S=\overset{n}{\underset{i=1}{\prod }}G_{i}$$

When all the s-a-0 and s-a-1 variables are set to false (0), the CNF formula *S *describes the good (fault-free) circuit behavior. The faulty circuit is a copy of the fault-free circuit, with faults (s-a-0 or s-a-1 variables) injected at the gates to be affected by faults.

We explain our approach using a simple example. Assume we are given the BN network from Figure 1, which has two gates *g*_1 _and *g*_2_, primary inputs *a*, *b*, *c*, and primary output *z*. Also assume and we want to model a stuck-at 1 fault on the output of gate *g*_1 _as shown in the figure. From our stuck at model, we insert an OR gate *g*_3 _at that location. We label the output of *g*_3 _as *e*, which is now an input to gate *g*_2_. The gate *g*_3 _has two inputs, *d *(the output of gate *g*_1_) and a side input *f*. With all inputs and outputs labeled, we obtain the CNF formula for each gates and the entire circuit.

$$\begin{array}{c} G_{\text{1}}=\;\left(\bar{d}+b+c\right)\cdot \left(d+\bar{b}\right)\cdot \left(d+\bar{c}\right)\\ G_{\text{2}}=\;\left(\bar{z}+a\right)\cdot \left(\bar{z}+e\right)\cdot \left(z+ā+ē\right)\\ G_{\text{3}}=\;\left(ē+d+f\right)\cdot \left(e+\bar{d}\right)\cdot \left(e+\bar{f}\right)\\ S=G_{\text{1}}\cdot G_{\text{2}}\cdot G_{\text{3}} \end{array}$$

The value of *f*, the side input to gate *g*_3_, determines whether the stuck-at 1 fault is activated or now. To activate the fault, *f *is set true by adding a clause (*f*) to the CNF, thus *S *= *G*1 · *G*_2 _· *G*_3 _· (*f*). Likewise, to deactivate the fault, *f *is set false by adding the clause $\left(\bar{f}\right)$ to the CNF, thus $S=G_{\text{1}}\cdot G_{\text{2}}\cdot G_{\text{3}}\cdot \left(\bar{f}\right)$. With our CNF formula for the circuit, we now describe several usage cases employing this CNF in SAT.

### Implementation of fault and drug simulation

#### Case 1: Single stuck-at fault identification

In this method, we find all single stuck-at faults which are non-redundant, as well as the faulty outputs that they generate. To proceed with this method, we first simulate the original circuit to determine the correct fault-free output. The circuit is simulated using our SAT formulation in the fault-free and drug-free model for a specified primary input value, and the resulting primary output value for the true response is saved as *Z*^0^.

The next step is to find all faults which are non-redundant. To avoid having to do an exhaustive search on all single stuck-at faults, we perform an All-SAT on the circuit *S *where we constrain the output to be not *Z*^0^. Assuming *n *output signals, this constraint is formed as the clause *C*^1^,

$$C^{1}=\left(\bar{Z_{0}^{0}}+\bar{Z_{1}^{0}}+\cdots \bar{Z_{n}^{0}}\right)$$

Here $Z_{i}^{0}$ is the variable corresponding to the *i^th ^*output bit.

Furthermore, we also add a constraint to *S *that the circuit contains only one fault that is injected at a time. This second constraint *C*^2 ^is formed by writing clauses of all pairwire combinations of faults, where *k *is the number of stuck-at faults and *f_i _*is the *i^th ^*fault.

$$C^{2}=\;\left(\bar{f_{1}}+\bar{f_{\text{2}}}\right)\cdot \left(f_{1}+\bar{f_{\text{3}}}\right)\cdots \left(\bar{f_{k-\text{1}}}+\bar{f_{k}}\right)$$

We now form a new CNF *S*^1 ^= *S *· *C*^1 ^*· C*^2^. The resulting All-SAT on *S*^1 ^is a list of all non-redundant single stuck-at faults and their faulty output. These faults are flagged for drug simulation using any of the next three cases.

The results from this case can also be used immediately in several ways. For example, this method classifies for each single stuck-at fault whether it is redundant or non-redundant. That is, any fault which is redundant does not produce an incorrect output, and can be ignored from a therapy standpoint. In a second example, the faulty output from the stuck-at model can be compared to a previously measured output from expression data, in order to identify which genes are potentially faulty. This information can be used to target genes for potential drug development, avoiding genes that are untestable.

#### Case 2: Fault rectification with fewest drugs

In the presence of a particular fault, the problem is determining whether a selection of drugs can rectify the circuit, i.e. change the faulty output to the correct output. If this is not possible, we want to obtain the "best" or "closest" output to the correct output, by using drugs. To do this, we guide the WPMS solver by assigning weights to the output states. For example, in the GF network used in our experiments, the fault-free output *Z*^0 ^is assigned the highest weight (80) and remaining output states are assigned decreasing weights (70, 60, 50, etc.) based on increasing Hamming distance (1, 2, 3, etc.) from the fault-free output. We assume that faulty states that have a larger Hamming-distance have a more pronounced cancer proliferative effect.

Additionally, the selection of drugs to achieve the best output should use the least number of drugs to minimize the side-effects on the patient. To incorporate this in the WPMS solver, each drug that is *not *selected is given a weight of 1. The GF network example has 6 drugs, thus if no drugs are selected, then the cumulative drug weight is 6. Likewise, if all drugs are selected, the drug weight is 0.

Note that the output and drug weights are assigned in such a way as to avoid the situation where a less-desirable output (with few drugs) is chosen over a higher weight output with more drugs. We assume that from a clinical standpoint, the priority is to first produce the best possible output, and secondarily to use the fewest drugs required for that output.

All faulty circuits with non-redundant faults from Case 1 are augmented with the output and drug weights and simulated using WPMS. The WPMS solver will implicitly and deterministically find the assignment of drugs that achieves the best possible output and with the fewest drugs. The output values, selected drugs, and highest weight of the fault+drug circuits are recorded and compared with the drug-free circuits. An immediate result from this method is that a fault where the fault+drug circuit which obtains its best output with zero drugs is in fact an *untestable fault*, wherein no drug combination can improve the output.

In general, several stuck-at faults can be simultaneously present in the circuit. A circuit with *n *lines can have 3*^n ^*- 1 possible stuck line combinations. This is because each line can be in one of the three states: s-a-1, s-a-0, or fault-free. All combinations (except one which has all lines in their fault-free state) are counted as faulty. In our implementation, multiple stuck-at faults can easily be modeled for rectification, by setting one or more lines to their faulty state.

#### Case 3: Fault rectification with minimal drug cost

In the previous case, all drugs are equal in terms of their weight. However, there may be a situation where we would want to differentiate the drugs based on some cost function based on characteristics such as price, number of side-effects, or ease of availability. For example, two drugs with few side-effects may be more desirable than one drug with many side-effects, if both drug selections produce the same output. As such, in the presence of a particular faulty circuit and desired output, the problem is determining a selection of drugs with lowest total cost.

Each drug that is not selected is given a weight proportional to its cost. In our example, we use the number of side-effects as the drug's cost. All faulty circuits with detectable faults from Case 2 are modified with the new drug weights. In addition, the output of the circuit is fixed to the best output as determined in Case 2. These circuits are then solved using WPMS to obtain the selected drugs with lowest cost.

#### Case 4: Determining therapy with fewest drugs and best coverage

From Case 2, we identify the drug selection that best rectifies a *certain *fault. However, in drug therapy, the fault location may be unknown. In this situation, a drug selection that rectifies all faults (or as many faults as possible) with the fewest drugs, is desirable.

For each faulty circuit (with a single fault), we find all combinations of 1, 2, and 3 drugs that yield the best output from Case 2. This is done by performing a WPMS All-SAT to find *all *satisfying drug selections with drug weight greater than or equal to *d *- 3, where *d *is the total number of drugs. Each drug selection (or vector) is analyzed to see how many testable faults are rectified or covered by it. The drug vector with the highest coverage and fewest drugs is recorded as a best candidate for therapy.

## Results

### Model implementation

We evaluate the WPMS-based ATPG methods on the GRN that models growth factor (GF) pathways [3]. In multicellular organisms, cell growth and replication is tightly controlled by the cell cycle control. This system receives signals from other cells which are used to decide whether the cell should grow. A failure in these signals can lead to unwanted or unregulated cell growth, leading to cancer. These signaling pathways are well studied, and several drugs have been developed to target different pathways for cancer therapy.

**Figure 3:**
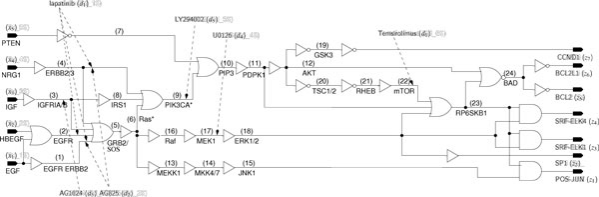
**Logic circuit stuck-at fault model for GF signaling pathways**.

We begin with a BN model of the GF pathways as derived in [3]. In this model, pathways are converted to an equivalent BN logic gate. Each interconnection (net) between logic gates is then assigned a numerical label. As stated in our approach section, defects in the GRN are represented as stuck-at faults that permanently set a signal net to 1 or 0. At each net, the logic gates for injecting a s-a-0 or s-a-1 are inserted. If there is a drug that targets the net, the appropriate logic gates are also inserted. The conversion of the faults and drug locations to a logic netlist is shown in Figure 3. The final circuit is then converted to CNF for further analysis.

In the results, stuck-at faults are referred by the net numbers that are affected (i.e. net 7 s-a-0, means that the signal corresponding to net 7 is stuck-at 0). The network has 5 primary input (PI) signals and 7 primary output (PO) signals. The PIs will be defined as a 5-bit binary vector *X *= [*EGF*,*HBEGF*,*IGF*,*NRG1*,*PTEN*], while the POs will be defined as a 7-bit binary vector *Z = *[*FOS *- *JUN*,*SP*1,*SRF - ELK*1,*SRF - ELK*4,*BCL*2,*BCL*2*L*1,*CCND*1]. In all tests, the PIs are fixed to *X *= 00001 as this input leads to the non-proliferative output in the fault-free case.

For this network, six drugs are available, defined as a 6-bit vector. Each bit corresponds to a drug, such that a value of 1 on the *i^th ^*bit indicates that drug *i *is selected, and a value of 0 indicates that drug *i *is not selected. The drug vector is *D *= [*lapatinib*,*AG*825,*AG*1024,*U*0126,*LY*249002,*Temsirolimus*].

All the methods (Case 1 through 4) were implemented using an open-source weighted partial Max-SAT solver called Maxsatz [12,13]. Our procedure consists of scripts which take the initial CNF, selects desired fault variables, sets output and drug weights, and solves the CNF using Maxsatz. The satisfying assignments are then parsed for the output and drug vectors, and reported in the results. In all examples listed in this section, the WPMS runtime was significantly less than 1 second per CNF.

### Simulation results

#### Case 1: Single stuck-at fault identification

In the single stuck-at fault model, each net was simulated for s-a-0 and s-a-1 with no drugs, and results compared with the fault-free circuit. For fault-free circuit with *X *= 00001, the output vector is *Z*^0 ^= 0000000. All single nonredundant stuck-at faults, which have an output different from the fault-free circuit, are recorded and shown in Table 1. In this table, the first three columns show the affected net, the stuck-at value, and the faulty output, respectively.

**Table 1:** Drug selection for single stuck-at faults

Net	s-a	Faulty PO	Best PO	Drug Vector	Score
1	1	1111111	0000000	010000	85
2	1	1111111	0000000	100000	85
3	1	1111111	0000000	001000	85
4	1	1111111	0000000	010000	85
5	1	1111111	0000000	000110	84
6	1	0000111	0000000	000110	84
7	1	0000111	0000111	000000	56
8	1	1111111	0000000	000010	85
9	1	0000111	0000000	000010	85
10	1	0000111	0000111	000000	56
11	1	0000111	0000111	000000	56
12	1	0000111	0000111	000000	56
16	1	0111110	0000000	000100	85
17	1	0111110	0000000	000100	85
18	1	0111110	0111110	000000	36
19	0	0000001	0000001	000000	76
20	0	0000110	0000000	000001	85
21	1	0000110	0000000	000001	85
22	1	0000110	0000000	000001	85
23	1	0000110	0000110	000000	66
24	0	0000110	0000110	000000	66

Each row in the table corresponds to a single fault, located by the net number and the type of fault (stuck-at-1 or stuck-at-0). The primary output [*FOS - JUN*,*SP*1,*SRF - ELK*1,*SRF - ELK*4,*BCL*2,*BCL*2*L*1,*CCND*1] shown is the expression value where 1 means the gene is expressed, while 0 means the gene is not expressed. The drug vector [*lapatinib,AG*825,*AG*1024,*U*0126,*LY*249002,*Temsirolimus*] indicates the drug selection where 1 means the drug is selected, while 0 means the drug is not used.

From this table, we observe that nets 13, 14, and 15 are not listed. The presence of a fault (s-a-0 or s-a-1) on these nets does not generate an incorrect PO, and as such, these are redundant faults. From a therapy standpoint, the genes corresponding to these faults can be ignored.

#### Case 2: Fault rectification with fewest drugs

From the results in Case 1, all non-redundant faults are simulated with drugs. The outputs are first weighted where the fault-free output *Z*^0 ^= 0000000 has a maximum weight of 80 as it represents a non-proliferative output. All remaining output vectors are given weights of 80 - 10*h*, where *h *is their Hamming distance from the fault-free output. The drugs are also given weights where the non-selection of a drug has a weight of 1. With six drugs, the maximum score is therefore 80 + 6 = 86.

Table 1 shows for each non-redundant stuck-at fault, the best output (Column 4), the drug vector to achieve such output (Column 5), and the weight score (Column 6). We observe that for many faults, there exists a drug vector that can completely rectify the fault, and produce a fault-free circuit. Additionally, the corresponding reported drug vector is minimal in the number of drugs used, which is desirable in therapy usage. We also determine that faults on nets 7, 10-15, 18, 19, 23, and 24 are untestable, as no combination of drugs can produce a change in the output. This can be explained as there are no drugs on the fan-out of these genes to rectify the fault.

To demonstrate the adaptability of our algorithm, we test it on a few examples of multiple stuck-at faults. Table 2 shows for a circuit with multiple stuck-at faults, the best drug selection for fault rectification (when possible). The columns of Table 2 have the same meaning as in Table 1.

**Table 2:** Drug selection for multiple stuck-at faults

Net	s-a	Faulty PO	Best PO	Drug Vector	Score
1,21	1,1	1111111	0000000	010001	84
4,9	1,1	1111111	0000000	000001	85
5,19	1,0	1111111	0000001	000110	74
6,8	1,1	1111111	0000000	000110	84
7,20	1,1	0000111	0000111	000000	56
8,21	1,0	0000111	0000000	000010	85
13,16	1,1	1111110	0000000	000100	85
1,3,6	1,0,1	1111111	0000000	000110	84
2,14,20	1,1,0	1111111	0000000	100001	84
4,7,17	1,1,1	1111111	0000111	010100	54
4,12,23	1,1,1	1111111	0000111	010000	55
8,9,11	1,1,1	0000111	0000111	000000	56
8,9,21	1,1,0	0000111	0000000	000010	85
12,18,20	0,0,0	0000110	0000000	000001	85
15,17,21	0,0,1	0000110	0000000	000001	85

Each row in the table corresponds to multiple stuck at faults, either 2 or 3 faults noted by the net number. The type of stuck-at fault is listed respectively.

#### Case 3: Fault rectification with minimal drug cost

When selecting drugs, there may be multiple drug combinations that may rectify a fault, but where each drug has a different associated cost. We first assign weights to drugs, according to their cost. For this paper, we use the number of side-effects as the drug's cost. Drugs AG825, lapatinib, Temsirolimus are assigned weights of 10, 15, and 35, respectively, which correspond to their approximate number of side-effects [14,15]. However, drugs AG1024, U0126, and LY294002 have yet to under go clinical trial and the number of side-effects is unknown. As such, these drugs are assigned a weight 20, which is an average of the 3 previous weights.

In this GF example, Case 3 simulation provides the same results as in Case 2. This is due to a lack of drugs that share paths in the circuit. In fact, for almost every non-redundant fault, the best output state can only be achieved through one drug vector.

#### Case 4: Determining therapy with fewest drugs and best coverage

Using the results from Case 2, we observe that the GF network has 13 testable faults. For these 13 faults, we perform an All-SAT to find the top three scoring drug combinations yielding the best output. All drug combinations are analyzed across all single faults and presented in Table 3 showing drug vector, count of faults rectified, and fault coverage. Drug vectors are ordered in increasing number of drugs selected.

**Table 3:** Drug selection count and fault coverage

Drug Vector	Count	Coverage	Drug Vector	Count	Coverage
**000001**	**3**	**23%**	**000111**	**13**	**100%**
000010	2	15%	001011	6	46%
000100	2	15%	001101	6	46%
001000	1	8%	001110	10	77%
010000	2	15%	010011	7	54%
**100000**	**3**	**23%**	010101	7	54%
000011	5	38%	010110	10	77%
000101	3	23%	011001	6	46%
**000110**	**10**	**77%**	011010	5	38%
001001	4	31%	011100	5	38%
001010	3	23%	100011	8	62%
001100	3	23%	100101	8	62%
010001	5	38%	100110	10	77%
010010	4	31%	101001	7	54%
010100	4	31%	101010	6	46%
011000	3	23%	101100	6	46%
100001	6	46%	110001	6	46%
100010	5	38%	110010	5	38%
100100	5	38%	110100	5	38%
101000	4	31%	111000	4	31%
110000	3	23%			

The drug vectors are grouped according to the number of drugs selected (1, 2, or 3 drugs). The count column notes the number of testable faults rectified by the drug selection, and the coverage shows the count as a percentage of total testable fault rectified. Bold rows indicate drug vectors with highest coverage for the number of drugs used.

From these results, we observe that with only 1 drug selected, the best coverage is only 23% of faults using lapatinib (*d*_1_) or Temsirolimus (*d*_6_). When allowing for 2 drugs, coverage increases to 77% using the drug combination of U0126 (*d*_4_) and LY294002 (*d*_5_). Finally, we achieve 100% coverage of all testable faults when using the 3 drug combination of U0126 (*d*_4_), LY294002 (*d*_5_), and Temsirolimus (*d*_6_). When the single stuck-at fault location is unknown, these selected drug combinations will be the most effective for therapy and for preventing the proliferation of cancer.

## Discussion

**Figure 4:**
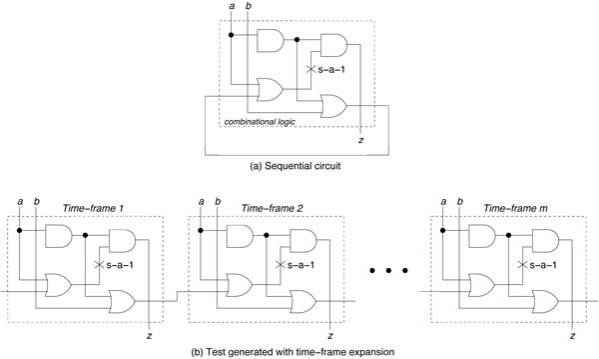
**Sequential ATPG by time-frame expansion method**.

In this section, we discuss the generalization of our approach to sequential circuits. Thus far, the SAT-based ATPG algorithm has been described for and performed on purely combinational circuits, wherein the primary output of the circuit is dependent only on the primary inputs. We observe that the output of the GF signaling pathway from the experiment is fixed based on the primary inputs, where the drug vector is technically also an input. In general though, the circuit representation of the BN can be sequential, where the primary output is determined by current state in addition to the input. The local GRN for mammalian cell-cycle [16] is one such example of a sequential circuit where gene expression updates based on the current gene state. If we consider a directed graph where the genes are nodes and edges are regulations upon other genes, then a combinational circuit (such as the GF signaling pathway) is acyclic. However, for a directed graph of a sequential circuit, a subset of genes will be inter-regulated forming directed cycles. As such, in the BN, a gene takes its current input (state of its regulatory genes and/or external inputs) and outputs a new state or value for the next time point. We assume in the BN that all genes update synchronously. In other words, for each primary input and current state, the resulting primary output and next state are determined for all genes, and that the next state becomes the new current state. While a synchronous update is biologically unrealistic, it allows us to have deterministic state transitions and simplifies the analysis for our ATPG algorithm. There are several methods for performing sequential ATPG, the most common of which is *Time-Frame expansion *[17]. As shown in Figure 4, the sequential circuit is replicated *m *times into a combinational circuit, which models *m *time steps of the sequential circuit behavior. The *i^th ^*copy is connected to the (*i *+ 1)*^th ^*copy such that the regulating genes from the *i^th ^*copy are connected to their target genes in the (*i *+ 1)*^th ^*copy. Each copy is called a frame, and additional frames can be added to the circuit for any length *m*. In this way, the sequential circuit is converted to a combinational circuit. After the conversion of the sequential circuit to a combinational *m *step expansion, we can apply our SAT-based ATPG algorithm. When we consider the fault-model of the circuit, we must assume the fault is persistent (i.e. the fault exists in all frames). The corresponding ATPG method must target multiple faults, or in other words, the same fault, but in different time frames.

One consideration for the sequential ATPG is the initialization of state in the first time frame. Ideally a known state should be used, such as one obtained from a previous microarray expression measurement. An alternative is to use an attractor state. In the long-term behavior, the dynamics of the BN transition to the attractors (attractor cycles), thus using an attractor state is a reasonable starting state for therapy.

The complexity of applying SAT-based ATPG to sequential circuits depends on the length of time-frame expansion. For a circuit with *k *variables in its SAT formulation, each frame increases the number of variables by *k*. The SAT search space is then 2*^km ^*for an expanded circuit with *m *frames. The number of frames for expansion can be bounded. If a subsequence of states has the same first and last state, then the sequence can be stopped. For a BN, the number of frames *m *can be bounded by the sum of the number of stesps it takes to reach an attractor cycle and the maximum length of the attractor cycles for all combinations of drugs under consideration. In the worst case, the number of frames required would equal to the number of possible states, which is 2^*n*+*d *^for a BN with *n *target genes and *d *drugs.

## Conclusions

In this paper, we have presented an efficient and extensible SAT-based ATPG methodology for cancer therapy. We approach this problem by representing the BN and cancer as a logic circuit stuck-at fault model. This circuit, along with the testing conditions, is converted into a CNF. The CNF is then augmented with output and drug vectors weights and solved using a weighted partial Max-SAT solver for four different usage cases: (1) single stuck-at fault identification, (2) fault rectification with fewest drugs, (3) fault rectification with minimum drug cost, and (4) determining therapy with fewest drugs and best coverage. We demonstrate these methods on the growth factor signaling pathway, and have presented results that are applicable to cancer therapy. While the GF network example in this paper is a combinational network, our algorithm can easily be extended to address sequential networks, like those found in transcriptional GRNs, by simply unrolling the sequential circuit in time and applying the same methods. Furthermore, all nets, inputs, outputs, and drugs can be assigned weights, which can be made variable, allowing the user to fine-tune the network or design therapies for any number of test situations.

## Competing interests

The authors declare that they have no competing interests.

## Authors' contributions

PKL developed the algorithm, designed and performed the simulations, and prepared the manuscript. SPK proposed the main idea, provided insights on interpretation of the algorithm and results, and helped on the manuscript. All authors read and approved the final manuscript.
